# In situ preparation of hetero-polymers/clay nanocomposites by CUAAC click chemistry

**DOI:** 10.3906/kim-2007-62

**Published:** 2021-02-17

**Authors:** Mehmet Atilla TAŞDELEN, Çağatay ALTINKÖK

**Affiliations:** 1 Department of Polymer Materials Engineering, Faculty of Engineering, Yalova University, Yalova Turkey; 2 Department of Chemistry, Faculty of Science and Letters, İstanbul Technical University, İstanbul Turkey

**Keywords:** Clay, click chemistry, nanocomposite, poly(ethylene glycol), poly(epsilon caprolactone)

## Abstract

A series of polymer/clay nanocomposites containing mechanistically two different polymers, poly(ethylene glycol) (PEG) and poly(epsilon caprolactone) (PCL), were prepared by simultaneous copper(I)-catalyzed alkyne-azide cycloaddition click reactions. Both clickable polymers, PEG-Alkyne and PCL-Alkyne, were simultaneously clicked on to azide-functional montmorillonite (MMT-N_3_) nanoclay to get corresponding PEG-PCL/MMT nanocomposites. The chemical structures of the resulting nanocomposites were verified by following azide and silicone-oxygen bands using FT-IR and characteristic bands of PEG and PCL segments using ^1^H-NMR spectroscopy. The combined XRD and TEM analysis confirmed that all PEG-PCL/MMT nanocomposites had partially exfoliated/intercalated morphologies. In addition, the increase of MMT-N_3_ loading not only improved the onset and maximum degradation temperatures of the nanocomposites but also their char yields. Furthermore, the incorporation of MMT-N_3_ in the polymer matrix did not significantly influence the crystallization behavior of both PEG and PCL segments.

## 1. Introduction

Polymer/clay nanocomposite has gained remarkable attention in recent years because the incorporation of nanoclay with polymer matrix leads to significant improvements in its mechanical and thermal properties when compared to the virgin polymer [1]. Due to their large surface area interacting with the polymer matrix, modification of the clays, distribution of clay layers in the matrix, and selection of preparation methods are key factors for polymer/clay nanocomposites. The clays are natural earth materials that are incompatible with organic polymers. In order to eliminate this limitation, the clays are commonly modified with quaternary ammonium salts ion-exchange reactions. This modification not only gives the clays organophilic character but also increases their layer’s distance; hence, polymer chains can easily diffuse the clay galleries and these layers can be homogenously distributed in the matrix [2]. There are three main methods for the preparation of polymer/clay nanocomposites, including solution exfoliation, melt intercalation, and in situ polymerization [3,4]. Among them, the latter method where clay layers are exfoliated during the polymer forming reaction is the most efficient method to obtain well-dispersed polymer/clay nanocomposites [5–13]. In recent years, a conceptually different approach has been developed by introducing polymer chains into the clay layers via click chemistry reactions as they bring a number of advantages, including high efficiency under mild conditions, and simple preparation set-up and purification steps [14–22]. In this way, clickable groups such as azide-alkyne [14–20], thiol-ene [23,24], thiol-epoxy [25,26], and maleimide-diene [27] partners can be integrated in either the clay surface or polymer chain-ends. The following click reaction such as copper(I)-catalyzed alkyne-azide cycloaddition (CuAAC), gives highly exfoliated polymer/clay nanocomposites, as it can be carried out with high efficiency under ambient conditions [28,29]. The CuAAC click reaction has also been applied for the preparation of different types of polymer nanocomposites using silica [30–32] and POSS [33–37] nanoparticles, carbon nanotube [38,39], and graphene [40,41].

A new concept that is based on simultaneous click reactions of two different alkyne-functionalized molecules in conjunction with an azide-functionalized molecules in a single step has been very recently reported [42–44]. Moreover, orthogonal combinations of two (or more) mutually exclusive click reactions allow the fabrication of complex macromolecular architectures as well as multifunctionalization of materials [45–60]. This strategy not only shortens the synthetic steps but also reduces the number of work-up, purification operations, and cost [61]. In this study, simultaneous CuAAC click reactions have been applied for the preparation of poly(epsilon-caprolactone)-poly(ethylene glycol)/montmorillonite in one-pot fashion. For this purpose, alkyne-functionalized PEG [62] and PCL [15] that were easily convertible into azide or alkyne functions were prepared according to literature procedures and subsequently clicked onto azide-functionalized MMT [14] clays via CuAAC click reactions. This simple strategy enables the attachment of the mechanistically two different polymers on to clay galleries, enabling the delamination of clay tactoids and formation of polymer/clay nanocomposites.

## 2. Materials and methods

### 2.1. Materials

Methanesulfonyl chloride (Acros 99.5%), epsilon caprolactone (CL, Aldrich, 97 %), ethanol (Acros 96%), *N, N, N’, N’’, N’’’* -pentamethyldiethylenetriamine (PMDETA, Aldrich, 99%), dichloromethane (Aldrich, HPLC grade), and triethylamine (Aldrich, HPLC grade) were purified by distillation just before use. Sodium azide (NaN3, Acros 99 %), tin(II) 2-ethylhexanoate (Aldrich 98%), copper (I) chloride (Cu(II)Cl, Acros, 97%), poly(ethylene glycol) methyl ether (mPEG, Aldrich, *M*_n_ = 5000 g/mol), propargyl bromide solution (C_3_H_3_Br, Aldrich, 80 wt. % in toluene), sodium hydride (NaH, Aldrich, 90%), and *N,N* - dimethylformamide (DMF, Aldrich HPLC grade) were used as received. Cloisite 30B as an organically modified clay, was purchased from Southern Clay products, Gonzales, TX, USA. Its organic content was determined by TGA and calculated as 21 wt. %. The organo-modified clay was dried under vacuum at 110 °C for 1 h before use. The azido-functional MMT-N_3[14]_, alkyne-functionalized poly(ethylene glycol) (PEG-Alkyne) [62], and poly(epsilon-caprolactone) (PCL-Alkyne) [15] were prepared according to previously reported procedures. 

**MMT-N**_3_: FT-IR (cm^-1^) = 3660 (free O-H), 2820–2980 (C-H), 2120 (N_3_), 1010 (Si-O); TGA (wt. loss %) = 26.2; yield: 0.78 g (78%, gravimetrically).

**PCL-Alkyne:** FT-IR (cm^-1^) = 2810–3000 (C-H), 1720 (C=O), 1470 (C-H), 1160 (C-O-C); ^1^H-NMR (CDCl_3_), *δ* (TMS, ppm) = 4.65 (s, 2H, CH2–C≡CH), 4.05 (m, CH_2_O on PCL), 3.70 (t, 2H, CH2OH), 2.45 (s, ^1^H, CH_2_–C≡CH), 2.35–2.25 (m, CH_2_C=O on PCL), 1.65–1.55 (m, CH_2_ on PCL), 1.40–1.35 (m, CH_2_ on PCL), *M* n,GPC= 5500 g/mol, Đ = 1.38; yield: 3.43 g (67%, gravimetrically).

**PEG-Alkyne:** FT-IR (cm^-1^) = 2940–2780 (C-H), 1462 (C-H), 1284 (C-H), 1099 (C-O-C); ^1^H-NMR (500 MHz; CDCl_3_; δ, ppm)= 2.45 (t, -C≡CH), 3.40 (s, -CH_2_CH_2_OCH_3_), 3.65–3.75 (br, -CH_2_CH_2_O-), 4.20 (d, -CH_2_C≡CH); *M* n,GPC= 5200 g/mol, Đ = 1.26; yield: 4.61 g (87%, gravimetrically).

### 2.2. Preparation of the PCL/PEG heteropolymers-MMT nanocomposites by CuAAC click chemistry

Azide-montmorillonite clay (1%, 3%, 5%, and 10% of the polymers by weight) and the same weight of PEG-Alkyne and PCL-Alkyne (0.11 g, 0.02 mmol) were dissolved in (2 mL) DMF in a round-bottomed flask and stirred. The required Cu(I)Cl (3.96 mg, 0.04 mmol) and PMDETA (8.36 µL, 0.04 mmol) were added into the mixture and then heated in an oil bath at 50 °C and mixed overnight. The obtained polymers were precipitated into methanol and then filtered, dried, and weighed.

### 2.3. Characterization

Thermal gravimetric analysis (TGA) was performed on Perkin–Elmer Diamond TA/TGA with a heating rate of 10 °C min under nitrogen flow. The powder X-ray diffraction (XRD) measurements were performed on a Siemens D5000 X-ray diffractometer equipped with graphite-monochromatized Cu Kα radiation (λ = 1.5405 A˚). TEM micrographs were obtained with a Philips CM100 apparatus using an acceleration voltage of 100 kV. Ultrathin sections (ca. 80 nm thick) were cut at –100°C from 3 mm thick hot-pressed plates using a Reichert-Jung Ultracut FC4E microtome equipped with a diamond knife. Because of the large difference in electron density between silicate and polymer matrix, no selective staining was required.

## 3. Results and discussion

**Figure 1:**
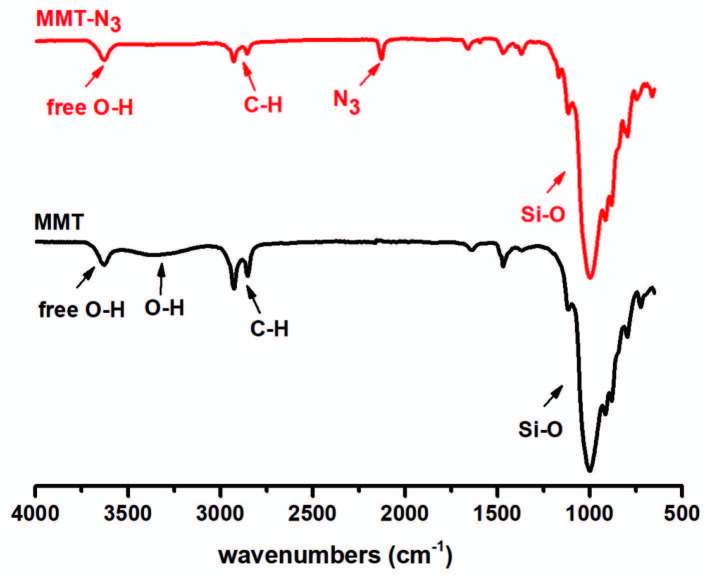
FT-IR spectra of MMT- (Cloisite 30B) and MMT-N_3_.

The use of click chemistry reactions for the preparation of polymer nanocomposites is a promising tool, providing high conversion under mild conditions and site-specific functionalization in easily removable or benign solvents. Among them, the CuAAC is the foremost utilized click reaction with unique properties of azides and alkynes, and the resulting triazoles. Therefore, it has been widely employed in both homogenous and heterogeneous conditions with solvent and substrate insensitivity. In the CuAAC, a clickable azido group can be simply incorporated by nucleophilic substitution reactions of halide- or hydroxyl-functionalities with sodium azide. In our case, hydroxyl-functionality of commercially available organomodified montmorillonite (Cloisite 30B) was firstly converted into the methanesulfonate group by treating with methanesulfonyl chloride. Subsequently, this methanesulfonate group was reacted with sodium azide to obtain the desired azido-functional montmorillonite (MMT-N_3_). This modification procedure was followed by FT-IR and XRD spectroscopies and thermogravimetric analysis. According to Figure 1, the characteristic O-H band at 3450 cm^-1^ gradually disappeared, while the azido band clearly appeared at 2120 cm^-1^. 

In addition, the d_001_ spacing for MMT-N_3_ initially presented at 4.87° shifted to 4.71° after nucleophilic substitution reaction. This change increased the *d* 001 spacing of clay layers from 1.81 to 1.87 nm. Furthermore, weight loss of was slightly increased from 21.1% to 26.2% after modification. The difference of 5.1% might be due to the attachment of azido groups that increase the organic content of MMT clay. On the other hand, the clickable PCL-Alkyne and PEG-Alkyne were synthesized by well-known ring-opening polymerization and nucleophilic substitution reactions. Their structures were confirmed by FT-IR, ^1^H-NMR, and GPC techniques (See experimental part). Finally, these clickable polymers were simultaneously attached onto the MMT-N_3_ surfaces by CuAAC click reactions to delaminate the stacked clay layers in the matrix. The fine distribution of clay layers on the nanometer level may lead to formation of nanocomposites with improved properties (Scheme).

**Scheme 1:**
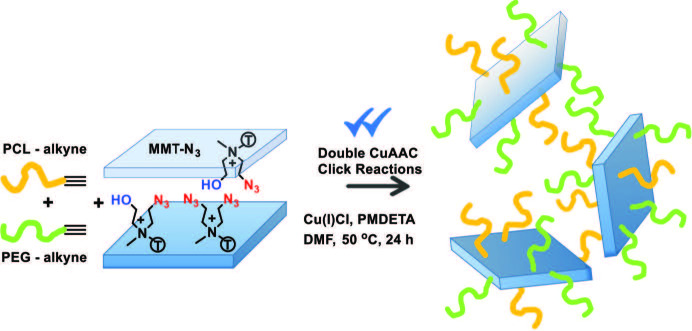
In situ preparation of PEG-PCL/MMT nanocomposites by simultaneous CuAAC click reactions.

**Figure 2:**
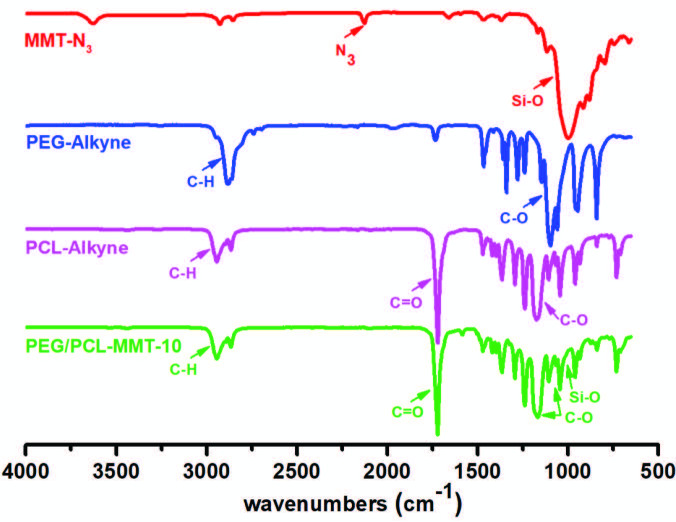
FT-IR spectra of MMT-N_3_, PEG-Alkyne, PCL-Alkyne, and PEG-PCL/MMT-10 nanocomposite.

The chemical structure of resulting nanocomposites was confirmed by comparing with spectra of their components; neat MMT-N_3_, PEG-Alkyne, and PCL-Alkyne using FT-IR spectroscopy (Figure 2). The characteristic peak of azide functionality of MMT-N_3_ at 2120 cm^-1^ clearly disappeared after the CuAAC click reactions. In addition to this, the typical siloxane, ether, and ester peaks of MMT-N_3_, PEG-Alkyne, and PCL-Alkyne were also verified at 1099, 1720, and 1160 cm^-1^, respectively. Overall, the disappearance of the azide band as well as the presence of siloxane, ether, and ester bands confirmed that the nanocomposite consisted of MMT, PEG, and PCL components.

**Figure 3:**
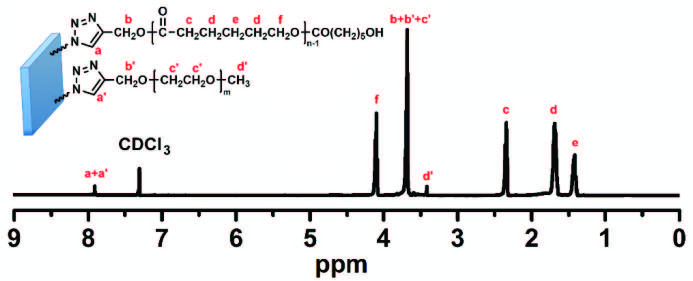
^1^H-NMR spectrum of PEG-PCL/MMT-1 nanocomposite.

In order to support these data, the chemical structure and polymer composition of PEG-PCL/MMT-1 was examined by ^1^H-NMR analysis (Figure 3).The successful synthesis of PEG-PCL/MMT-1 nanocomposite was confirmed by the appearance of new peaks belonging to PCL repeating units at 4.29 ppm (Hf) (repeating unit CH_2_O(C=O)), 2.31 ppm (Hc) (repeating unit O(C=O)CH2), 1.64 ppm (Hd) (repeating unit O(C=O)CH_2_CH_2_CH_2_CH_2_CH_2_O(C=O)), 1.38 ppm (He) (O(C=O)CH2CH2CH2CH2CH2O(C=O) and PEG repeating units at 3.71 ppm (Hc’) (repeating unit CH2O)in the spectrum (Figure 2b). In addition, a new peak belonging to the triazole ring was also observed at 7.97 ppm. Furthermore, the composition of PEG-PCL/MMT-1 was also calculated from integration ratios of specific resonances (Hc’ proportioned with Hc + Hd + He + Hf) belonging to both PEG and PCL segments. According to this calculation, the molar content of the PEG was found to be 46%, while it was 54% for PCL.

**Figure 4:**
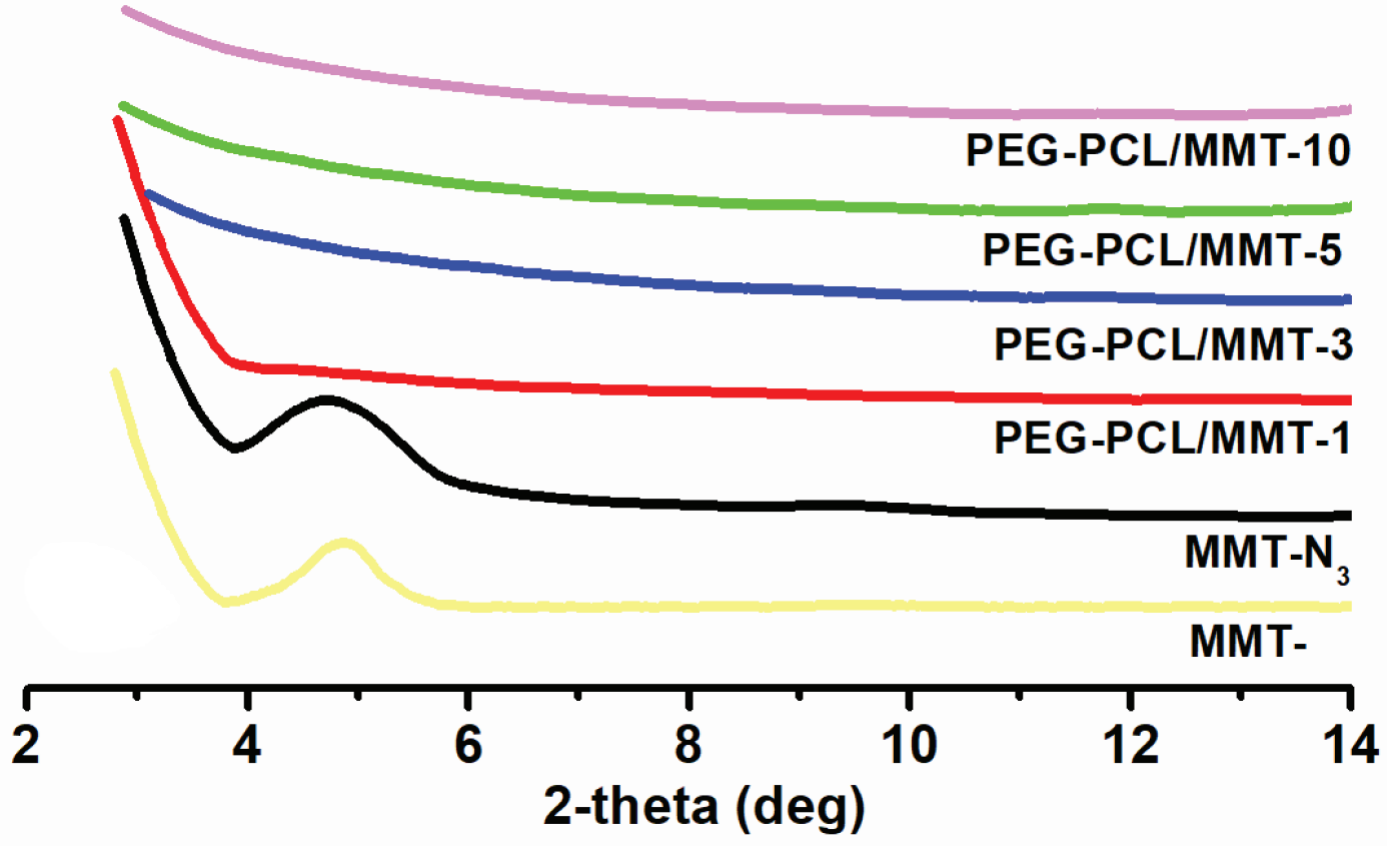
XRD graphs of MMT-, MMT-N3, and PEG-PCL/MMT nanocomposites with different MMT-N_31H-NMR spectrum of PEG-PCL/MMT-1 nanocomposite._ loadings (1%, 3%, 5%, and 10%).

XRD analyses of the PEG-PCL/MMT nanocomposites with 1, 3, 5, and 10 wt. % nanoclay loadings were performed to determine their crystal structures and compared with initial MMT-N_3_. The strong and sharp crystalline peak at 4.71° was attributed to a basal space of 1.87 nm coming from the crystallographic planes of silicate layers of MMT-N_3_. After the CuAAC click reactions, this peak completely disappeared in all PEG-PCL/MMT nanocomposite samples. The absence of a diffraction peak implied that all silicate layers were likely to be exfoliated in the polymer matrix (Figure 4). 

**Figure 5:**
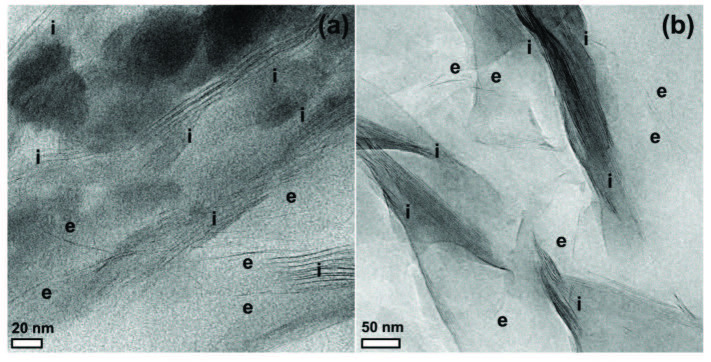
TEM micrographs of PEG-PCL/MMT-1 in low (scale bar: 50 nm) and high (scale bar: 20 nm) magnifications (e and i assigned to exfoliated and intercalated layers).

The similar results for the polymer/clay nanocomposites prepared by CuAAC click reaction were also reported in the literature [15,19,20]. However, the XRD analysis alone may not be a conclusive tool in determining the exfoliated clay plates, especially at low concentration. Therefore, the use of TEM analysis was crucial to provide a better understanding of the morphology of the nanocomposites. In order to get more detailed information on the exfoliation and dispersion of individual galleries, TEM analysis with two different magnifications as displayed in Figure 5 for PEG-PCL/MMT-1 sample was carried out. In these micrographs, dark lines represented cross-sections of the individual silicate layers, whereas the brighter area displayed the polymer matrix. From the TEM observation, it appeared that mixed morphologies consisting of exfoliated and intercalated structures (highlighted by e and i) existed. The coexistence of mixed exfoliated/intercalated morphologies might be explained by a competition between polymer diffusion and clay delamination. The limited mobility of polymer chains as well as high surface energies of clay layers kept them tight rather than dispersed in the polymer matrix. Overall, both XRD and TEM analyses verified mixed exfoliated/intercalated morphologies in the PEG-PCL/MMT nanocomposites.

**Figure 6:**
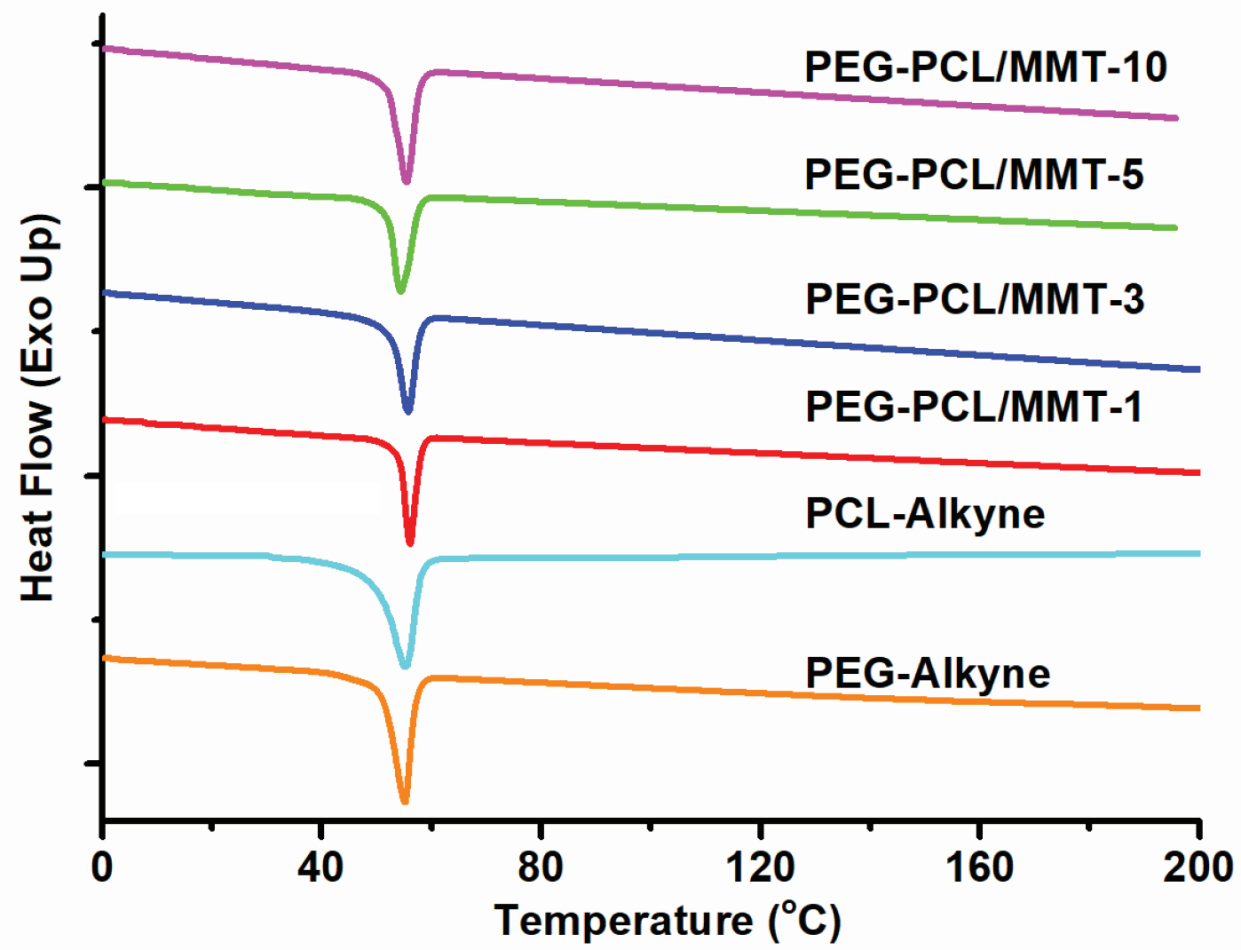
DSC thermograms of PEG-Alkyne, PCL-Alkyne, and PEGPCL/MMT nanocomposites with different MMT-N_3_ loadings (1%, 3%, 5%, and 10%).

Thermal properties of PEG-PCL/MMT nanocomposites were also investigated by both DSC and TGA measurements. The melting temperature ( *T* m) of PEG-Alkyne and PCL-Alkyne were measured as being very close to each other at 54.7 and 57.5 °C. After the nanocomposite formation, the *T* m values of all nanocomposites were detected between those of PEG-Alkyne and PCL-Alkyne. Furthermore, a slight increase was also recorded on the *T* m of related nanocomposites by increasing MMT-N_3_ loadings. Consequently, the formation of nanocomposites did not significantly influence the crystallization behavior of the both PEG and PCL segments (Figure 6).

**Figure 7:**
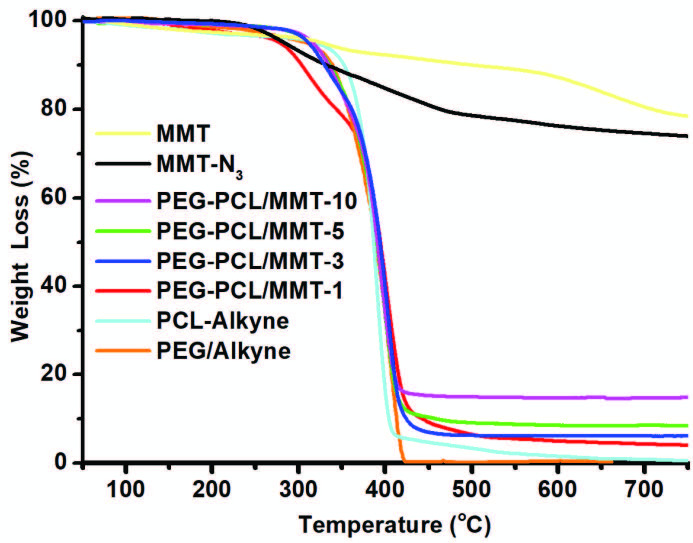
TGA thermograms of MMT, MMT-N_3_, PEG-Alkyne, PCLAlkyne, and PEG-PCL/MMT nanocomposites with different MMT-N_3_ loadings (1%, 3%, 5%, and 10%).

The onset ( *T* 10%) and maximum ( *T* 50%) weight loss temperatures, and the char yield were summarized in Table. It can be seen that all precursors and nanocomposites displayed a single-step degradation between 320 and 450 °C (Figure 7). This degradation could be due to the depolymerization, removal of low molecular weight compounds and side-groups, and random chain-breakage of the polymer chains. The incorporation of MMT-N_3_ not only improved the *T* 10% and *T* 50% temperatures of neat PEG and PCL but also remarkably increased those of the nanocomposites. Furthermore, the char yields of nanocomposites were higher than that of neat polymers and slightly increased by clay loadings under nitrogen atmosphere. These thermal improvements could be due to the barrier effects of nanoclay that hinder the diffusion of gas in the nanocomposites.

**Table:** Physical properties of PEG-PCL/MMT nanocomposites and their components for comparison.

Entry	d_001_^a^(nm)	T_m_^b^(°C)	T_10%_^c^(°C)	T_50%_^c^(°C)	Char yield^c^(%)
MMT-	1.81	-	570	-	79.9
MMT-N_3_	1.87	-	256	-	74.8
PEG-Alkyne	-	54.7	328	389	-
PCL-Alkyne	-	57.5	338	388	-
PEG-PCL/MMT-1	n.d.^d^	54.9	333	394	2.0
PEG-PCL/MMT-3	n.d. ^d^	55.3	341	397	4.8
PEG-PCL/MMT-5	n.d. ^d^	55.5	344	400	9.6
PEG-PCL/MMT-10	n.d. ^d^	55.6	353	404	18.7

^a^ Basal spacing (d_001_) is calculated by XRD analysis. ^b^ determined by DSC and analyses under a nitrogen flow at a heating rate of 10 °C/min. ^c^ onset (T_10%_) and maximum (T_50%_) weight loss temperatures determined by TGA analysis under a nitrogen flow at a heating rate of 10 °C/min. ^d^ probably complete exfoliated nanocomposites.

## 4. Conclusion

In conclusion, a series of PEG-PCL/MMT nanocomposites with different nanoclay loading were prepared in situ using CuAAC click reactions. The simultaneous attachments of PEG-Alkyne and PCL-ALkyne chains onto MMT-N_3_ not only triggered the exfoliation of clay plates but also enabled the formation of nanocomposites. The chemical, morphological, and thermal properties of the resulting nanocomposites were explored by FT-IR, ^1^H-NMR, XRD, TEM, DSC, and TGA analyses. The presence of MMT nanoclay and fulfillment of the CuAAC click reactions were confirmed by following the characteristic Si-O and azide bands using FT-IR spectroscopy. The chemical structure of the resulting nanocomposite was also verified by following characteristic bands of PEG and PCL segments using ^1^H-NMR spectroscopy. The molar content of the PEG was found to be 46%, while it was 54% for PCL in PEG-PCL/MMT-1 nanocomposite. The nanocomposites with mixed exfoliated/intercalated morphologies were evidenced in both XRD and TEM investigations. According to DSC and TGA analyses, the thermal properties of the nanocomposites were higher than those of neat PEG-Alkyne and PCL-Alkyne. This simple route will open a new avenue to prepare the multifunctional polymer nanocomposites using mechanistically different kind of polymers.
